# Necrology

**Published:** 1857-03

**Authors:** 


### NECROLOGY.

HUGH MILLER.—The mournful and tragical end of Mr. Hugh Miller, of Edinburgh, is probably already known to most of our readers; but the following more particular account of the circumstances attending the sad event will be read with much interest:

"Science has to mourn the loss of one of its brightest ornaments, and the learned ranks of Edinburgh will not easily replace the void occasioned in them by the death of Mr. Hugh Miller. Last Wednesday week the painful intelligence of Mr. Miller's death rapidly became known, and an announcement to that effect, made at the *Witness* Office, confirmed the sad news. For some time past, it appears, Mr. Miller had been a victim to extensive cerebral disease. Excessive study and midnight labor—the worst species of mental exertion—induced even *his* mind to succumb, and the calamity of his death to follow as the result. Latterly, a moody apprehension that his mind was giving way had seized upon him; he was a prey also to the idea that he was a somnambulist, and nightmare, in its most violent form, so frequently attacked him as to render the body more wearied upon rising in the morning than it had been the previous evening. Professor Miller saw him the day before his death, and came to the conclusion that extreme mental exertion had weakened his whole system, affected his digestive organs, and threatened serious head affection. Prescribing a total discontinuance of work, early hours, and slight diet, Professor Miller and Dr. Balfour doubted not that their directions, if carried out, would substantially relieve the patient. But it was too late. Already cerebral disease had formed, and the severe paroxysms it had inflicted on him, rendered him during the interval unconscious, and entirely irresponsible for his actions. On the morning of Wednesday week, the body of Mr. Miller, half dressed, was found lying lifeless on the floor of his study. A bullet from a revolver had pierced the left lung, grazed the heart, cut through the root of the pulmonary artery, and lodged in the rib of the right side. Near at hand, a sheet of paper, inscribed with burning words, told too truly of the insane impulse which had induced the deed, and taken from Scotland one of her most zealous sons in the cause of science. The name of Hugh Miller is essentially linked to the science of his adoption—geology. With the Old Red Sandstone, especially, his name is inseparable. His own vivid powers of description, combined with his intimate acquaintance with his subject, have made his works pre-eminent above all others on geology of recent publication. His 'Footprints of the Creator, or the Asterolepis of Stromness,' intended to expose what he deemed the fallacies of the 'Vestiges of the Natural History of Creation,' is, perhaps, the most elaborate contribution the world received from his pen. There is one source of gratification left, melancholy though it be: his last great work, 'Testimony of the Rocks,' upon which, for the last few months, he put forth all his energies, and with such terrible results, was finished, and will soon come before the world, now invested with a sad and peculiar interest. 'I have finished it this day,' he said, with marked satisfaction, to Professor Miller, on the day before his death; and deplorable as the event is, yet it cannot fail to soften the anguish of his friends when they recollect that Hugh Miller was permitted to finish his great work, which he especially intended should harmonize the doctrines of science with Bible truths. It was the last epoch of a great life, well in unison with his sound religious views, Christian spirit, and kindly bearing. At the early age of fifty-four, Hugh Miller has been taken from the world he so adorned; but long will the memory of his genial disposition and kind nature remain green in the memory of those who knew him. Long will his friends remember each memento of the past, and zealously retain all they may possess of their connection with him. Those who knew him will, with the writer, now doubly feel the value of that knowledge, and hoard carefully all that may remain to them of Hugh Miller."—*Lancet.*

---

DR. JOHN A. PARIS.—Died, in London, on the 24th of December last, Dr. John A. Paris, President of the College of Physicians, and one of the most eminent members of the English profession. Dr. Paris was born at Cambridge, in 1785, and therefore passed the usual limit of man's life. Graduating in medicine at an early age, he devoted his long life with an intense zeal to his profession; and even at the ripe age of threescore and ten, took upon himself the laborious duties of President of the Medical Council of the Board of Health, and with his own hand wrote the introductory report on the cholera of 1854. He was actively engaged in the practice of his profession in London for more than forty-five years, and held the honorable position of President of the College of Physicians since 1844.

"Dr. Paris was not only known as a physician of the highest eminence—he was as remarkable for his literary ability. The life of Sir Humphry Davy will ever remain one of the classical biographies of the English language. In connection with Mr. Fonblanque he also wrote the Medical Jurisprudence, which has remained a text-book with lawyers until our own day. His works of a more professional character were his treatise on Diet, which first brought him into notice, and which was published at a very early age; his Pharmacologia, which has run through more editions than most books; and his work on Medical Chemistry. Besides these and many other publications, his Philosophy in Sport has attained an enormous popularity, and with his life, the motive for an incognito, which was never really maintained, has altogether terminated. In so brief a notice as the one to which we are necessarily limited by considerations of space, we can say but little more.

"The last ten days of Dr. Paris's life were spent in the midst of excruciating sufferings, which were borne with the most remarkable fortitude. His chief concern appeared to be to console and comfort those around him, who could ill disguise their grief at the impending and irreparable loss. His intellect remained to the last as clear as at any time of his life, and while power of speech remained, nobody who listened to him could believe that the end was so near at hand. The public and the medical profession have suffered great loss in the death of John Ayrton Paris, one of the most disinterested, honorable, and able men who have ever practised the profession of medicine. The grief of his own family, and of those whom he honored with his friendship, is not matter of public concern, save in so far as it may serve to show how this wise and good man was honored and beloved by those who knew him best."—*London Paper.*

